# Hospital at Home – an emerging opportunity for internal medicine trainees

**DOI:** 10.1016/j.fhj.2024.100198

**Published:** 2024-10-26

**Authors:** Stephanie Moore, Rebekah Schiff, Daniel S. Furmedge

**Affiliations:** a Department of Ageing & Health, 9th Floor North Wing, St Thomas’ Hospital, London, United Kingdom; b Centre for Ageing Resilience in a Changing Environment, Faculty of Life Science and Medicine, King's College London, London, United Kingdom

**Keywords:** Internal medicine training, Medical education, Hospital at home, Virtual ward, Internal medicine

## Abstract

Hospital at Home (HAH) is growing at pace in the UK and is an acute clinical service that takes staff, equipment, technologies, medication and skills usually provided in hospitals and delivers that hospital care to selected people in their homes or in nursing homes. Services are as yet mostly excluded from doctors’ core training rotations. In parallel, non-traditional training pathways are being created to allow doctors to meet competencies outside of higher specialty training programmes. Evaluation of working in HAH as part of a locally designed internal medicine equivalence programme was undertaken, with specific focus on whether internal medicine curriculum competencies could be obtained. HAH provides valid internal medicine training, offering unique opportunities in clinical decision making, risk management, multidisciplinary team working, palliative medicine, and core internal and geriatric medicine. Reduced exposure to specific procedural skills and resuscitation in HAH can be mitigated across a balanced training programme. As HAH services develop, inclusion of HAH within internal medicine training programmes is recommended, alongside further research into their potential as a training environment.

## Highlights

• Hospital at home offers an innovative and unique training environment for learning generalist skills.
• Internal medicine curriculum competencies can be obtained through rotation to hospital at home as part of a balanced training programme.
• Hospital at home offers improved exposure to older adults and geriatric syndromes, immersion in community healthcare, advance care planning and end of life care, risk management and shared decision making, leadership and teamworking skills.
• With an increasing focus on community healthcare, internal medicine training programmes should routinely include hospital at home.

## Introduction

The UK internal medicine curriculum was revised and implemented in 2019 with a renewed focus on generalist skills.^1^ There is mandated exposure to geriatric medicine, critical care, acute unselected medical admissions, outpatient clinics and palliative medicine. There was a shift from presentation-based competencies to acquisition of generic and clinical competencies (Table 1) in practice, with a heavy focus on hospital-based inpatient and outpatient activity. In parallel, the Department of Health and Social Care has placed hospital at home (HAH) high on its priority list, with a national rollout taking place at pace.^2^^,^^3^ These services are defined by their ability to provide healthcare to people who would otherwise require an acute hospital bed. At present in the UK, the prevalence, structure and delivery of HAH are varied.^3^^,^^4^ Also called virtual ward, the UK Hospital at Home Society has called for use of united terminology of ‘Hospital at Home’. Accordingly, this paper will use HAH rather than virtual ward.

**Table 1:** Learning outcomes – capabilities in practice IMT stage 1 curriculum.

Learning outcomes – capabilities in practice (CiPs)
Generic CiPs
1. Able to successfully function within NHS organisational and management systems 2. Able to deal with ethical and legal issues related to clinical practice 3. Communicates effectively and is able to share decision making, while maintaining appropriate situational awareness, professional behaviour and professional judgement 4. Is focused on patient safety and delivers effective quality improvement in patient care 5. Carrying out research and managing data appropriately 6. Acting as a clinical teacher and clinical supervisor
Clinical CiPS
1. Managing an acute unselected take 2. Managing an acute specialty-related take 3. Providing continuity of care to medical inpatients, including management of comorbidities and cognitive impairment 4. Managing patients in an outpatient clinic, ambulatory or community setting, including management of long-term conditions 5. Managing medical problems in patients in other specialties and special cases 6. Managing a multidisciplinary team, including effective discharge planning 7. Delivering effective resuscitation and managing the acutely deteriorating patient 8. Managing end of life and applying palliative care skills

Guy’s & St Thomas’ NHS Foundation Trust (GSTT) launched its HAH service in 2012. It now has over 100 ‘beds’ in the London boroughs of Lambeth and Southwark. Patients are referred from multiple sources including the ambulance service, community teams and the acute hospital. Medical staffing in the service has expanded from GPs and consultant geriatricians, to include other postgraduate doctors from foundation year 2 to senior clinical fellow. The service accepts referrals for adults ages 18 years and older with a wide range of medical problems. The most common conditions seen are infection, heart failure and chronic obstructive pulmonary disease, geriatric syndromes such as falls and delirium, and people approaching the end of life.

An innovative year-long programme of geriatric medicine rotations for clinical fellows was developed, which included a 4- or 6-month HAH rotation. The remainder of the programme was spent in inpatient geriatric medicine wards, acute frailty unit, research and medical education. With a renewed focus on alternative training routes, GSTT has also developed an alternate pathway for locally employed doctors to achieve internal medicine (IMT) stage 1 training. Doctors on this pathway complete rotational training identical to an IMT programme using the same curriculum, portfolio and requirements, but with local governance and performance review. Sign-off is via the alternative certificate to enter group 1 or group 2 specialty training. As a conscious decision, it was decided to include a 4-month HAH rotation within this programme as one of the six rotations over the 2-year programme.

This qualitative local service evaluation aimed to answer the following questions:

• Can HAH provide appropriate educational experience to meet the IMT curriculum requirements as part of a wider rotation?
• What opportunities and experiences could an HAH rotation offer, above and beyond the experiences of a traditional hospital-based IMT stage 1 programme?
• What areas of the IMT stage 1 curriculum would an HAH rotation not be able to deliver?

## Method

This work aims to describe the experience of a single HAH service and was performed as part of a service evaluation registered at GSTT. All data collected were anonymised in line with Caldicott principles and formal ethics board approval was not required. This was a service evaluation. All those who completed the questionnaire did so with consent.

A questionnaire was designed to identify the alignment of HAH experience with IMT generic and clinical capabilities in practice (CiPs), as well as the acceptability of integration of HAH into IMT training. It employed both quantitative and qualitative data collection. The questionnaire was distributed by email to all 27 junior clinical fellows who worked in the GSTT HAH service between 2020 and 2024 as part of a geriatric medicine fellowship or an IMT equivalent year.

Responses were collated and data were subsequently analysed. Quantitative outcomes were summarised using descriptive statistics including frequency with corresponding percentages, while qualitative outcomes were analysed using thematic analysis with comments coded, grouped and merged to identify themes.^5^^,^^6^

## Results

The response rate was 89% (n=24/27). Of those who responded, most (n=18) were either undertaking or planning to undertake IMT stage 1 training. The remaining six respondents’ pathways were general practice (n=2), radiology (n=1), public health (n=1) and other non-training roles (n=2).

The majority (79%, n=19) agreed that HAH was a suitable training rotation, with 75% (n=18) interested in pursuing this area following completion of training. For those who did not agree (n=5), only one had pursued IMT training. The majority (75%, 18/24) would be interested in an HAH role following completion of training.

Analysis of responses to identify advantages and disadvantages of HAH for training are summarised in Table 2.

**Table 2:** Summary of core benefits and disadvantages around hospital at home work.

Advantages	Disadvantages
Management of individual people • Holistic, person-centred care • Heart failure management • Medication administration • Rationalising and justifying use of investigations • Frailty, older adults and comprehensive geriatric assessment • Shared decision making • Advance care planning, palliative medicine and end of life care experience • Breadth of internal medicine	Less exposure • Fewer procedural skills • Less exposure to resuscitation • Less acute unselected take • Less outpatient clinic • Fewer opportunities for teaching and research
Teamwork skills • Working within a multidisciplinary team • Improving communication skills	Supervision • Perceived less supervision than a hospital-based role
Leadership skills • Clinical independence including development of clinical skills and acumen • Managing risk and uncertainty	
Understanding of NHS systems and structure • Understanding of community services • Understanding of healthcare resource • Population, demographic understanding	

Qualitative analysis identified five key themes with regards to the benefit of HAH for IMT rotations: improved exposure to older adults and geriatric syndromes, increased community experience, enhanced exposure to palliative care, advance care planning and end of life care, with greater opportunities for independence, decision making, shared decision making and risk management and the chance to develop leadership and teamworking skills.

### Improved exposure to older adults and geriatric syndromes

While unwell older adults are often experienced in the hospital environment, reviewing them in community settings added a whole breadth of experience and exposure.

*‘… elderly care is mandatory in IMT … [HAH] adds another dimension … a real opportunity to prevent admission … to independently prescribe and practice clinical communication skills.’*

*‘Felt good hands*-*on experience developing you as a clinician’*

### Increased community experience

Being in a different environment allowed the focus of medicine to be different and broadened perspectives of doctors on the environments that populations are living in.

*‘Seeing the homes/care homes that you will be discharging people to, as a hospital clinician in the future, really develops your understanding/perspective of what is important to do prior to discharge.’*

### Advance care planning, palliative and end of life care

Significant exposure to people approaching the end of their lives was key, particularly decision making in different settings and having the time to think more about future care. This made doctors feel more confident and able to make decisions independently.

‘… *supporting patient choices to be at home for their treatment when hospital not their preferred option/unlikely to benefit them’*

*‘The most memorable and fulfilling experiences I had during my time there were when we helped people to stay at home with their family’*

### Decision making, shared decision making and risk management

HAH allowed doctors to upskill in taking risk, seeing what was possible in a community environment and in particular having conversations and sharing risk with patients and their representatives. Important was the notion that this is more challenging in more rigid, scaffolded hospital settings.

*‘I developed skills in being able to assess a patient … without seeing the patient, which will be valuable when having to act as medical registrar’*

*‘It allowed for excellent practice in the delivery of patient-centred care with a focus on shared decision making’*

### Leadership and teamworking skills

Being in an environment with less direct supervision, including doing independent home visits, led to needing to take a lead more. A collective teamworking approach was highlighted as essential with the patient out of direct sight most of the time.

*‘I had never worked so closely within an MDT setting before* – *this really helped my teamwork and leadership skills.’*

The curriculum areas are less well delivered through an HAH rotation are outlined in Table 1. Of note, an outpatient community falls clinic now forms part of the rotation and community-based procedures such as ECG, bladder scanning and intravenous medication administration are all procedural skills practised in the HAH rotation.

## Discussion

With the ever-emerging importance of generalism at the top of the national agenda, HAH provides a vehicle within this training programme where many trainees will go on to be medical registrars and then work long term in hospital-based specialist roles.^7^ The opportunity to work in a community setting where clinical decision making and shared decision making were some of the highlights seems too good an opportunity to miss. New consultant roles in many specialties are changing, with increasing numbers expected to support HAH and other community work. Given that the definition of HAH includes secondary care clinical expertise, the imperative to train doctors to work in these settings is already present.

That some generic CiPS (Table 2) were highlighted as being covered in more depth was reassuring, including communication, ethical and legal issues and understanding of NHS organisational issues related to integrated care. These are issues not always well taught across an IMT programme, and HAH provides an opportunity to experience these in some depth in a different setting. There was felt to be good coverage of clinical CIPS 4 (community setting and long-term conditions), 6 (multidisciplinary team working and discharge planning and 8 (end of life care and palliative medicine).

Key areas of the curriculum that were felt to be less well covered were management of the acute unselected take. Over 50% of the referrals to the GSTT HAH service are acute referrals from general practice, community teams, care homes and the London Ambulance Service. Though presentations seen in HAH may vary to those seen on a hospital-based acute unselected medical take, this does not equate to a lack of educational opportunities.

A lack of outpatients experience can be resolved in one of two ways; a clinic can be included, or community experience can be prioritised with additional outpatient clinic experience balanced in another rotation, highlighting the importance of a whole-programme approach and oversight by programme directors.

Limited procedural exposure was highlighted as a downside to HAH. It is important to recognise that feedback at the IMT Specialty Advisory Committee is that procedural skills are not always accessible in hospital-based rotations either. Programme directors therefore need to address how this could be delivered regardless of time spent with HAH. There is an abundance of procedural experience in HAH, but these procedures are not always aligned to the core IMT procedural skills. Increasing skills among internal medicine doctors, particularly those trained in acute internal medicine, points to an evolving role for point of care ultrasound and ultrasound-guided procedures to be within the gift of HAH services as they develop.

Managing the acutely unwell patient is certainly a skill required in HAH, but inevitably opportunities for delivering advanced life support, critical care referrals and resuscitation will be less. It is important, however, that skills in managing critically unwell patients not for escalation with different treatment options gives a different perspective, important when most hospital inpatients are frail older adults needing clear treatment escalation decisions. Escalation is still required when patients need readmission to hospital and in the trust at which this evaluation related, in-hospital cardiac arrest rates are less than two per month, again highlighting that just being in hospital does not always provide the experience needed. Other methods like simulation are increasingly used to provide experience in cardiac arrest.

The balance of each IMT rotation is different and it is the whole programme that is required to develop and acquire all of the IMT curriculum competencies. Careful planning of the whole programme can therefore mitigate these concerns to ensure balance. HAH can contribute to the delivery of the palliative medicine requirement in IMT training.

## Limitations

There were a number of limitations associated with this evaluation. This was a local evaluation which reviewed one advanced service (the GSTT Hospital at Home service) in one geographical area (Lambeth and Southwark). There is limited generalisability and extrapolation ability from the data, especially in view of the vast differences in makeup of HAH services across the UK. The majority of those who had completed the rotation were doing it as part of a geriatric medicine junior clinical fellow year, so views may be biased towards those more inclined to enjoy HAH and community work and less representative of the general cohort doing IMT training. This evaluation was also only able to address the first level of Kirkpatrick’s model for evaluation of a training programme ‘level 1: Reaction – evaluate the learner’s reactions and responses to training’.^8^

## Conclusion

HAH offers a new and unique learning environment for internal medicine training, offering a breadth of relevant experience in both internal and geriatric medicine outside of the traditional hospital environment. Particular areas of opportunity are development of clinical decision making and risk management skills, multidisciplinary team working, palliative and end of life care and better understanding of, and immersion in, community services. As HAH services develop further, internal medicine training programmes should routinely look to rotate IMT doctors through a community rotation such as HAH, carefully placed to ensure balance of the IMT curriculum across the programme.

## Data statement

The data that support the findings of this service evaluation are available by contacting the corresponding author.

## CRediT authorship contribution statement

**Stephanie Moore:** Writing – review & editing, Writing – original draft, Methodology, Formal analysis, Data curation. **Rebekah Schiff:** Writing – review & editing, Writing – original draft. **Daniel S. Furmedge:** Writing – review & editing, Writing – original draft, Supervision, Methodology, Formal analysis, Data curation, Conceptualization.

## Declaration of competing interests

Rebekah Schiff reports a relationship with UK Hospital at Home Society that includes: board membership. Daniel Furmedge reports a relationship with NHS England London that includes: employment. If there are other authors, they declare that they have no known competing financial interests or personal relationships that could have appeared to influence the work reported in this paper.
